# Challenges and Solutions Surrounding Environmental Enrichment for Dogs and Cats in a Scientific Environment

**DOI:** 10.3390/ani11102980

**Published:** 2021-10-15

**Authors:** Emma Desforges

**Affiliations:** Waltham Petcare Science Institute, Freeby Lane, Waltham-on-the-Wolds, Leicestershire LE14 4RT, UK; emma.desforges@effem.com

**Keywords:** effective enrichment, canine, feline, animal welfare, individual, sensory, social stimulation

## Abstract

**Simple Summary:**

Environmental enrichment is the provision of equipment or enhancement to an animal’s living area with the goal to improve animal welfare. Equipment is often provided without assessment of suitability or continuous revision. Enrichment constraints can vary between different animal settings and include limited space, budget and lack of opportunity for enhancement. Simple improvements and attention to the animal’s individual needs and preferences can allow further enrichment optimisation.

**Abstract:**

Dogs and cats housed in research-, kennel- and cattery-type settings are reliant on caregivers to optimise their day-to-day experiences and welfare. The goal is to provide enriching environments for physical, social and environmental control; behavioural choice and opportunities to live as varied a life as possible. However, there are numerous challenges in these environments such as lack of appropriate enrichment for group housing, budget for equipment/training, study controls, time and space to make improvements. In addition, research settings are required to comply with legislation for care, husbandry and housing, and as standards differ between regions, conditions will vary between settings. Sharing knowledge in this field can only help drive a wider culture of care by helping improve the lives and welfare of animals cared for. This article presents some of the environmental enrichment strategies effective at the Waltham Petcare Science Institute, UK.

## 1. Introduction

Government regulations and scientific guidance documents detailing the minimum standards for the care and accommodation of animals exist [1,2,3]. The requirement for environmental enrichment in UK research settings is recommended in the Code of Practice for the Housing and Care of Animals Bred, Supplied or Used for Scientific Purposes [4], though specific details such as quality, benefit, type, frequency of exposure, etc., are lacking. Furthermore, enrichment provision is not listed as a mandatory requirement, only that it should enhance exercise and cognitive activity as well as being tailored to the species. This ambiguity allows research settings to be flexible in their enrichment delivery, which can both benefit and compromise animal welfare.

The Waltham Petcare Science Institute, UK is a global research and development site for Mars Petcare. The housing for 200 cats and 200 dogs has welfare at the highest priority and aims to exceed regulatory standards for enrichment and care. 

Environmental enrichment can provide physiological, psychological and social enhancement and allow opportunities for expression of species-typical behaviours [5,6,7,8]. However, delivering optimal enrichment and environmental design can present conflicting challenges in a research setting, where there is a need to balance any impact of enrichment on data quality and control. Additional challenges such as budget, material durability, foreign body risks, enrichment rotation and sensory limitations add to the complexity. Here, examples of enrichment solutions that exist at Waltham are shared, with the aim that these could be of value to dogs and cats housed in other settings. 

## 2. Value of Enrichment

The environment an animal lives in can significantly impact its welfare. Environmental enrichment strategies for dogs and cats in research, kennel and cattery settings are widely reported, and it is generally accepted that opportunities for physical, social and environmental control should be provided [6,7,8,9,10]. Enrichment equipment is abundant for domestic use, yet resources are rarely designated for non-domestic purposes, where it is typically required to be more robust and durable. Regular wet cleaning and multi-animal use can result in a higher turnover of equipment and in high financial investments being made in enrichment equipment that is not safe, fit for purpose or does not meet the intended goal. Measured choices are needed to ensure any budget spent has a good return of investment and avoids the need for frequent repurchasing. Identifying the enrichment benefit and risk to the animal by piloting the use of the equipment is a useful initial approach to assess whether the enrichment is of the expected value. This evidence of benefit can further help influence budget holders and drive other welfare initiatives. Risks of any physical harm to the animal can include foreign body ingestion, injury caused by equipment damage or resource conflict with another animal; usually, all are avoidable if adequate quantities of equipment are provided and assessed for supervised/unsupervised use. Equipment auditing can help safeguard against human health and safety risks and foreign body/trauma risks but also to measure use; if it is being underutilised and of no value to the animal, it should be removed, replaced or relocated elsewhere. At Waltham, new equipment is piloted over a 2–4-week period depending on the item (toy or furniture) and with multiple cats, dogs and breeds. Feedback is collected from the caregivers who aim to balance the risk and benefit. One example of a suitability pilot was that of paddock shelters for dogs; pig pens and children’s playhouses were piloted and neither were deemed appropriate as they were either too warm in the heat, easily chewed or a human manual handling risk for cleaning. A further example was new wall-mounted shelving for cats which was well utilised by the cats but required considered height placement due to wall structure and head bump risks for people. Quality time spent during social interactions and health/grooming checks are a good opportunity for caregivers to become familiar with the individual’s personality and preferences. Varying rewards such as food, toys and human contact can help inform the caregiver on the different values the animal places on reinforcers and experiences and is a useful insight to enrichment tailoring.

## 3. Canine Enrichment

**Figure 1:**
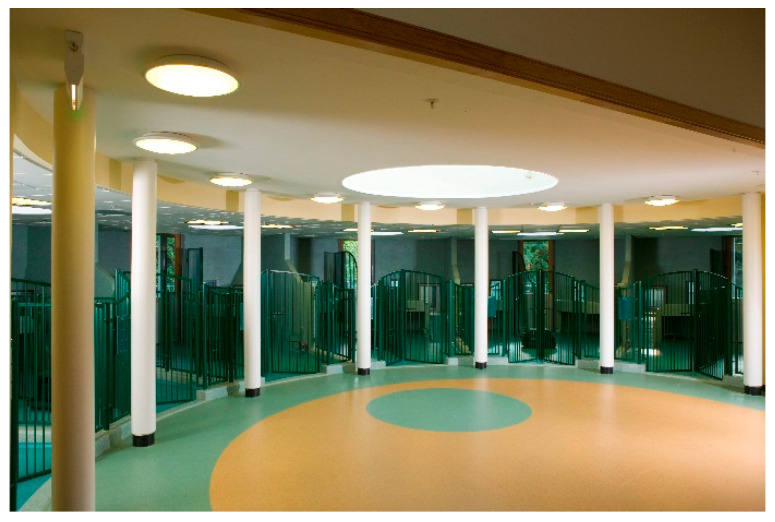
An example of a Waltham dog pod allowing the dogs both visibility and privacy between pens.

Dogs living at Waltham are housed in social pairs from puppies, each with access to an internal and external pen area and connecting outdoor grass, concrete, artificial grass and/or wet pour rubber paddocks. The pens are in pods, allowing the dogs both visibility and privacy between pens (Figure 1). The internal pens are mechanically heated, and all dogs have a raised bed and/or platform. Various bedding types are provided, tailored to the dog’s specific needs. Soft bedding such as Vetbed^®^ is well utilised, and the stainless steel frame Kuranda^®^ beds have proven to be relatively chew-resistant, durable and easy to clean. 

Paddocks are shared by 4–6 dogs in adjacent pens, with pairing and grouping of dogs carefully managed for compatibility, behaviour, age, health and neuter status. All dogs have access to the internal and external pens 24 h a day. Free access is provided to paddocks during the daytime. In addition, all dogs have time away from the pen and paddock during daily exercise on- and off-lead. This recreational time is essential for physical activity, mental stimulation and dog interaction and will vary each day per dog. Off-lead recreation is usually with small groups (4–6 dogs) and includes play, free roaming in enclosed fields and woods or agility activities for a minimum of 20 min per dog per day. Dogs have ad libitum access to mains-supplied water drinkers and are individually fed in the pen by temporarily partitioning the internal and external pens. The times and frequency of meals vary depending on the dog’s age and study requirements, but dogs are generally fed twice a day. 

The Code of Practice for the Housing and Care of Animals Bred, Supplied or Used for Scientific Purposes from December 2014 [4] states that the provision of platforms within dog pens offers several benefits, including increasing the complexity of pens, providing a viewpoint and sleeping/resting area for dogs on top of the platform and providing a retreat or sleeping area under the platform. Hubrecht [11] found that dogs spent over 50% of their time on platforms, suggesting that platforms are a positive pen resource for laboratory-housed dogs. When provided with a platform, the dogs no longer had to rear onto their hind legs to view out of the pen; however, barking increased. The dogs in the study had minimal human interaction, visibility or time outside of their pen and were housed in a spatially and enrichment-restricted environment. These limitations would likely have impacted the behavioural measures observed as well as the value and use of the platform. Approximately 50% of the pens at Waltham have fixed ramp-accessible platforms (raised area within the pen). Whilst some dogs do consistently utilise the platform area as a vantage point for visibility in and out of the pen during the day, very few dogs sleep or rest on the platform. Dogs that have both a raised platform with bedding and a contained bed on the pen floor generally choose the latter for sleeping and resting. Unless the pen is adequately sized, the platform can have the negative impact of reducing space and limiting opportunities for conspecific resting and interaction. Dogs are a highly social species, and numerous studies have shown that dogs choose to spend time in physical contact with kennel mates [12,13] and that separation from a conspecific negatively affects behaviour and stimulates the immune system [14]. Observations within the Waltham dog population concur with Doring et al. [15] that soft beds are preferred over plastic beds, and the dog’s preference is for comfort rather than elevation. The benefit of pen platform provision must be balanced against the risk. No reduction in animal welfare has been observed in dogs at Waltham that do not have access to pen platforms. although the dogs are in a resource-enriched environment. In some cases, the provision of the platform has become problematic. The resident veterinary team have requested that dogs <1 year or with pre-existing mobility issues are housed in pens without ramps/platforms where possible to minimise any risk of joint trauma or disease caused by jumping off the platform area. The pen size does not always allow for a gradual, progressive ramp or steps, and literature on the impact of specific step height or ramp gradients on differing ages or breeds is lacking. Until this area is scientifically clearer, activities which encourage jumping from a height or abrupt movements, such as jumping off a platform, are avoided where possible. This recommendation adds complexity to housing management, where housing of dogs to appropriate pens can conflict with suitable pen availability and colony stability. Consequently, permanent platform installation in new facility developments has been reconsidered and replaced with removable raised areas, thus enabling a more individualised enrichment approach. 

Toys are an essential enrichment for play, to relieve mild frustration, to combat boredom and to direct chewing [16,17]. Inappropriate chewing can be classified as biting or chewing which causes trauma (damage to teeth or mouth) to the individual and destruction to the environment. This behaviour can result from medical etiology stress and environmental or social factors. In kennel environments, the damage caused through inappropriate and destructive chewing can have significant animal welfare and financial implications. Chewing directed at the environment can be identified by chew damage to the pen, beds, door frames and handles, enrichment and flooring. In contrast, acceptable chewing is deemed to be chewing or biting which is directed at chew-approved toys or naturally occurring items without causing trauma to the individual. Food and chew toys can help manage unacceptable chewing and other behaviours, though the choice of chew toy should be considered to manage any health risks posed to the dog through the use of hard chew [18,19]. 

At Waltham, toys have been selected based on handler experience, durability, the activity/behaviour they were designed to target (e.g., chewing or cooperative play), dog age, size and suitability for supervised/unsupervised access. Toy rotations occur at least twice a day to keep items novel, encouraging dogs to elicit play and exploratory behaviours. Soft, fabric toys are washed weekly in a washing machine whereas hard toys that might damage the machine are cleaned with disinfectant spray. Novel presentation such as freezing toys and hiding in sandpits, around paddocks or in cardboard boxes or other destructible enrichments provides variety and increases the toy appeal. During training, a box containing a mixture of hard and soft toys, chews, cardboard and odorous material can be presented to the dog for them to choose their reinforcer. This is especially useful when a toy’s value can change over time and also informs the caregiver on the dog’s preference. This choice of reinforcer is highly rewarding for the dog and can help deliver positive interactions with the environment, situation and handler. 

Alternative food presentation means such as slow or puzzle feeders and snuffle and lick mats are especially enriching where focus and methodical puzzle-solving skills are required. Hiding/scattering food in the dog’s environment and encouraging it to “find it” creates an enriching and slower mealtime compared to standard or slow feeder bowls and can be considered for specific dogs who benefit from the mental challenge. 

**Figure 2:**
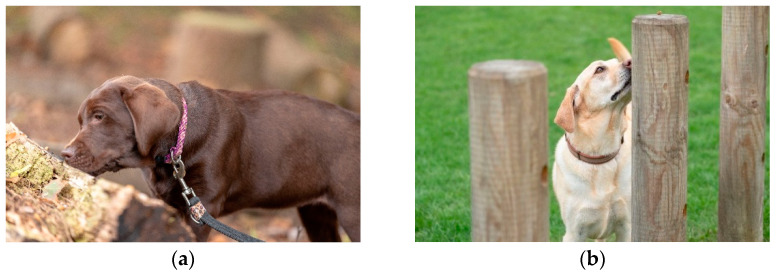
(**a**) A dog at Waltham having a slow walk through a wooded area. (**b**) A dog at Waltham experiencing scent play during an outdoor social session.

Sensory enrichment is one of the simplest, economic and stimulating forms of enrichment and often the most underutilised. Auditory, olfactory and visual methods of sensory stimulation can be easily applied to internal and external environments and can help reduce arousal-related behaviours [20,21]. A dog’s olfactory system is highly developed, and presentation of new and novel odours is especially enriching and a valuable tool for dogs less motivated by toys or food. Scent work or scent play can be both a reinforcer and a trained behaviour to dogs of all ages. Teaching dogs to use their nose to find toys, food or people can help anxious dogs by building confidence, or excited dogs to channel their energy into a calm activity which is mentally tiring. Simple and effective ways to provide opportunities for sniffing include using snuffle mats and hiding food in rolled-up towels or scrunched-up paper stuffed into boxes. Hiding a ball in long grass or slowing a walk down allows the dog the opportunity to sniff or dictate the route of a walk by following its nose (Figure 2a). Even areas that are familiar to the dogs will have an ever-changing odour, whether it is from people, cleaning regimes or other animals. 

Scent work can be incredibly useful during training, grooming, health examinations or sample collection. Scent depositories such as material/carpet tile samples used during training/handling sessions can divert dogs from displacement sniffing to targeted sniffing where they are encouraged to go and follow their nose (Figure 2b). This can help the dog’s engagement in a session and can also be used as a distraction and confidence builder. Scent work training classes are widely available and a good Continued Professional Development (CPD) investment with principles that can be readily applied to multiple settings.

Paddock enrichment is provided in multiple forms to enhance active, exploratory, sensory, play and social behaviours. Wooden enrichment structures lack durability from weathering and chewing and are high-maintenance. The life cycle and environmental impact of enrichment have resulted in most wooden structures being phased out and replaced with durable and recycled plastic furniture. Although an expensive initial outlay, the furniture does not rot, corrode or splinter and is therefore considered a good return on investment. Paddock equipment and placement has been tailored to breed, age and benefit, with dog breeds consistently showing clear preferences for enrichment types, generally typical to breed traits [22,23,24]. The Terriers prefer chasing and tunnelling activities whilst the Beagles utilise the height and visibility from raised areas. Labradors show a preference for more floor-based enrichment such as pallets for resting, paddling pools and digging activities. Awareness of breed-typical behaviours and preferences can help with tailoring to meet individual dogs’ needs. Despite this being a challenge within a shared external space, it is achievable by providing variety and choice. Rotating equipment use across areas is an effective way of adding environmental and sensory variation. 

**Figure 3:**
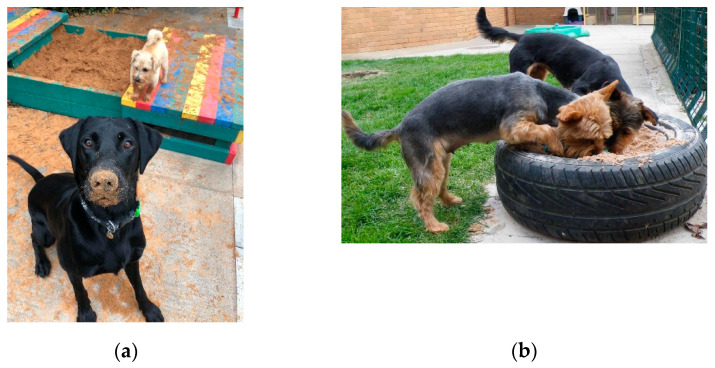
(**a**) Dogs having playtime in a Marmax sandpit with a sliding cover at Waltham. (**b**) Dogs interacting with a sand-filled tyre at Waltham.

Digging is a natural behaviour for dogs and may be genetic for some breeds or observed and copied by conspecifics [25,26]. Dogs at Waltham will frequently dig holes in grass paddocks, which has health and safety implications as the digging unearths potential foreign body objects (such as stones) and creates holes and trip hazards for staff. Observations of dogs at Waltham showed that the provision of the sandpit (containing child-safe play sand BS EN 1177) in dog paddocks reduced the frequency of digging in the grass areas as well as a total overall decrease in the quantity and weight of paddock debris collected. Sandpits with covers have the benefit of keeping the sand clean and dry and can be used as a resting area when covered, although sandpits can also be made using containers or tyres (Figure 3a,b). As the dog population at Waltham is consistent and paddock groups stable, the sand does not require regular changing or disinfection between use. However, an alternative dig-and-find opportunity could be achieved by providing a cardboard box filled with shredded paper and toys. 

## 4. Feline Enrichment

Cats living at Waltham are housed in social groups of approximately 10 cats from kittenhood. As with dogs, groups are carefully managed for compatibility and sociality according to behaviour, age, health and neuter status. Internal cat rooms have free access to indoor conservatories or an external enclosed area called a “catio”, and all cats have human and intraspecific socialisation in a separate play area. Cats have visibility to the outdoors and to internal spaces, including other cat rooms. Internal cat rooms are mechanically heated with some heated shelf areas. Both floor- and wall-based enrichment is provided, including towers, scratch posts, beds, toys and hiding opportunities. Litter trays are positioned at least 0.5 m apart from other resources, and a minimum of one litter tray per two cats is provided. This differs from the guide of one litter tray per cat plus an additional one extra [27,28], but may be adequate in a multi-cat setting where cat groups are generally stable and part of the same social group. If elimination occurs outside of the litter tray, additional trays are provided; however, this needs to be managed carefully in a group setting so as not to limit the placement of other essential resources. In order to meet individual preferences, litter trays vary in size and design (open, covered, low/high-sided, front/side/top entry) and are distributed in separate fixed locations around the living area. Access to mains-supplied water drinkers is ad libitum, and cats are individually fed in feeding areas either within or external to their living area. The times and frequency of meals vary depending on the cat’s age and study requirements, but cats are generally fed twice a day. Individual access via microchip cat flaps to provisions such as food or litter trays can allow the collection of individual data from cats housed in their normal social setting without the need for single housing.

Group housing of cats can present enrichment challenges and opportunities [29,30]. Enrichment choice, quantity and location can impact intraspecies sociality, and it is essential that resources allow adequate social distancing expression of individual behaviours [31]. This is especially necessary in group settings where there are mixed personality and age profiles. A shared living space does not have to result in shared resources and can be achieved through considered placement and space use. Taking the time to observe the dynamics and behaviours within the group is essential to identify any gaps in enrichment equipment or placement. Cats often show preferences for locations and furniture, and to help retain environmental stability, key resources such as beds, hiding areas, marking aids and litter trays should not be regularly moved or changed [32,33]. Provision of hiding opportunities (such as a cardboard box) can help reduce stress and the cat’s ability to cope by avoiding interactions [34,35]. Pheromonal marking is normal and can be supported by providing multiple scratch areas, grooming aids and surfaces for olfactory signals (carpet/cloth). Cats have a complex sensory system relying on chemical and olfactory communication such as urine spraying and pheromonal marking, which is used to establish boundaries and maximise their sense of security and comfort. A cleaning regime which retains some olfactory familiarisation and continuity is therefore important for maintaining a sense of security [36]. At Waltham, daily dry spot cleaning is performed to remove excess debris (fur and litter) off surfaces, and bedding is only replaced if soiled or dirty. Target cleaning is completed on an as-needs basis and concentrates solely on cleaning and disinfection of a specific, localised area (such as a soiled area). A wet deep clean is completed every 1–2 weeks (as required), which involves a more thorough change, cleaning and disinfection of the entire area. Changing and cleaning of litter trays are dependent on the litter substrate; however, soiling is removed at least three times a day.

**Figure 4:**
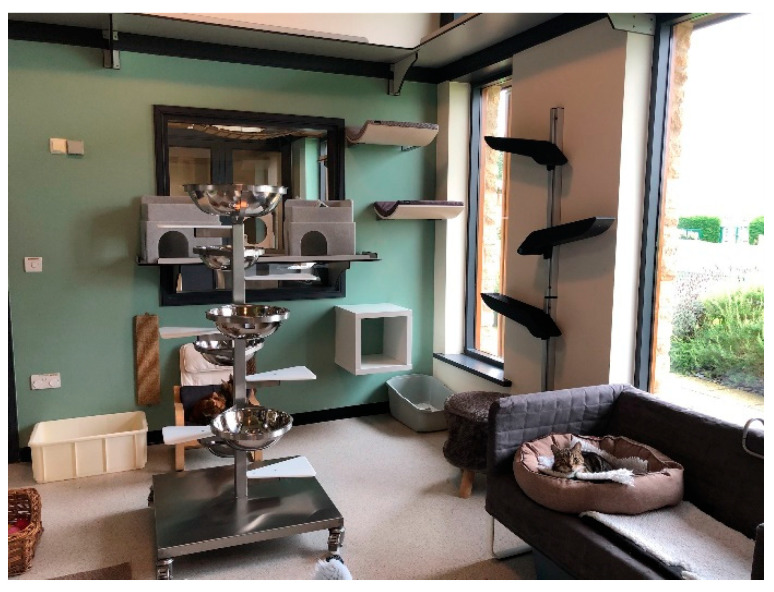
A cat housing room at Waltham showing a stainless steel bowl cat tower and use of walls for enrichment.

Beds provide comfort, resting and hiding resources which are of high importance for cats, being a fundamental piece of equipment that can affect how a cat or a group of cats settles and interacts within its environment. Bedding type is varied by material, function and location with at least one bed per cat. This reduces competition for key resources but still allows for cats to rest together if they choose to do so by providing a variety of bedding types; beds where the cats can partially or fully hide and wide and long beds for spreading out and/or sharing are tailored to the individual cat’s preferences. Placing the bedding at different heights and locations allows the cats some choice over their environment [26,37]. High shelf-, wall- and window-mounted beds are observed to be used more than floor- or low-level beds, and high shelves can be reached for cleaning using extendable, angled cleaning tools [37]. Wooden shelving has been gradually replaced with Trespa^®^ due to its durability, impermeability and safe, non-slip surface for cats. The shelving is deep enough (around 300 mm) to allow for passing places and placement of bedding and hiding areas and has a small, intermittent 20-mm upstand to help retain bedding on the shelf. Using the three-dimensional space within the cat housing frees up the floor space to provide more opportunities for enrichment. As well as using perimeter wall space and height, towers are positioned more centrally within the cat rooms to provide vantage and passing places and spatial separation (Figure 4). Commercial cat towers are generally made of wood and designed for domestic settings. They are often unsuitable due to being difficult to clean and relocate or unable to withstand multi-cat environments. Previously used wooden or fabric towers needed frequent changing or repair, which was inefficient and uneconomic. Wooden towers are gradually being replaced with stainless steel for durability (Figure 4). Sisal rope for scratching and climbing can be wrapped and glued around steel columns and simply replaced when needed. The expectation is that the towers will last for many years and can be donated or recycled when no longer required. 

**Figure 5:**
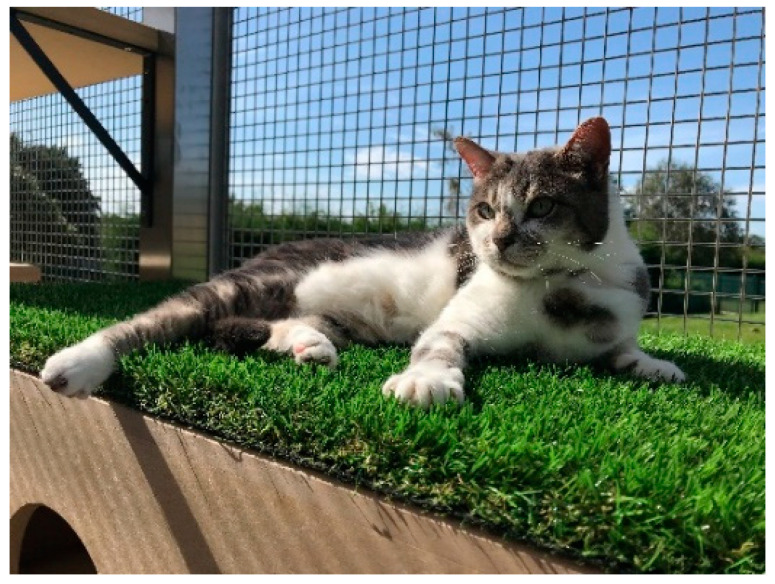
A cat resting on an AstroTurf-covered shelf in the enclosed catio area at Waltham.

Cats housed in research settings may have limited access to external elements, sensory stimulation and exercise opportunities. Additional socialisation areas separate to the housing rooms are provided to add variation in equipment and enrichment activities. Providing cats with safe exposure to outdoor elements is highly enriching for cats, delivering auditory, olfactory and visual sensory stimulation. Outdoor enclosures or secured, mesh windows allow cats to experience sounds, odours and weather and view new activities outside of their living environment [38] (Figure 5). 

Exercise can be encouraged through providing interactive play with caregivers between cats, toys, treat balls and equipment. At Waltham, cat wheels are provided in both supervised and unsupervised areas and used daily by around 40% of cats. The cats will spend 1–2 min at a time on the wheel by themselves or in groups of 2–3 cats. A cat wheel can be used to relieve frustrations or redirect energy and is a good exercise outlet for some cats; running is a behaviour many indoor cats seldom have the opportunity to perform. Some cats can find the movement or noise of the cat wheel in motion unsettling at first, which can be managed through gradual introduction and training. Using toys or food can help with creating a positive association with the wheel. 

Toys such as wands and string toys encourage positive interactions and active movement in both the cat and the handler. Small soft toys are well received by most cats who enjoy chase, play, catch and carry behaviours but, as with string toys, can require supervised play to prevent foreign body risks. It is sometimes the simplest enrichment that is most effective; a piece of paper or foil rolled into a ball, a plastic stick poking out from a piece of bedding or a child’s play tent. It can be beneficial to look beyond species-specific toys, especially if it means that more equipment can be provided for longer or unsupervised due to its durability. Children’s plastic play equipment is an economic form of enrichment, readily available and easy to clean and can be draped with beds and blankets to create areas for hiding and comfort. 

The effect of catnip (*Nepeta cataria*) has previously been reported to be elicited in around 60–70% of cats [39]. The catnip response is inherited, though not evident in kittens under 8 weeks of age, and may not develop fully until 12 weeks of age [32,40]. A recent study suggested that all cats respond to catnip expressed as active, passive or with a combination of responses [40]. Catnip stimulates a temporary pleasure or euphoria response in cats, lasting up to 15 min with a 1-h refractory period, where further stimulation with catnip will have no effect [39]. At Waltham, cats that do not engage with the other toys will generally interact with a catnip toy and become more playful for a time, especially when exposure to catnip has been absent for a certain duration. The use of catnip toys can enrich the environment, allowing cats to exhibit some of their natural behaviours, and is an economic form of environmental enrichment.

**Figure 6:**
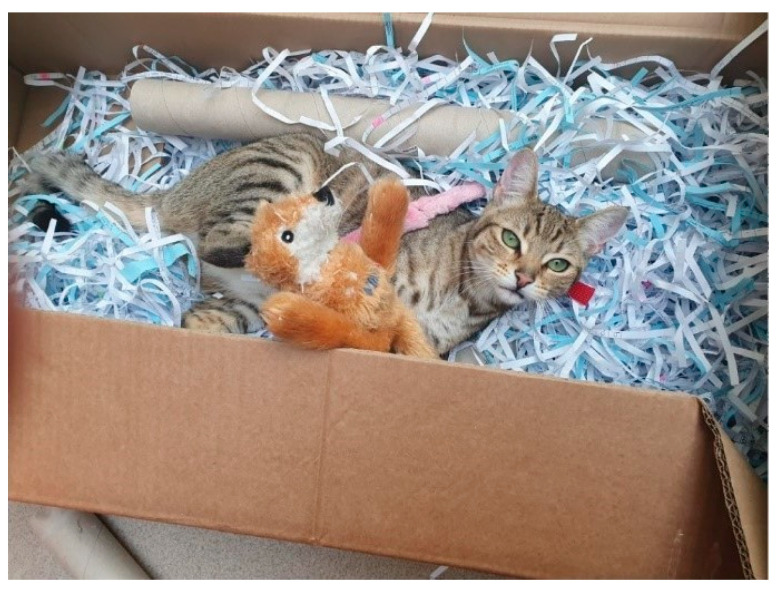
A cat at Waltham enjoying enrichment in a toy-filled cardboard box.

Some cats can find training stimulating, and games such as clicker, nose or paw targeting can be highly engaging [41,42]. Frequently changing or randomising toy presentation can help retain the cat’s interest and toy engagement, though some cats choose not to participate in human social interactions and prefer to rest, observe, explore or play by themselves. Having awareness of individual preferences but still providing opportunities and choice for these cat behaviours is essential [43,44]. For example, allowing the cat to choose a novel toy from a selection offered in a box or bag encourages the cat to explore, sniff and search for something they want, activating the seeking system, or they may just choose to play or rest in the box (Figure 6). 

## 5. Conclusions

Provision of appropriate enrichment in research-, kennel- and cattery-type settings can be challenging. Concept piloting can help demonstrate benefits to stakeholders and drive positive change. Increasing the variety of enrichment options available through height, materials, indoor and outdoor access, toys and sensory stimulation can help provide dogs and cats with environmental choice. 

A positive relationship between the animal and the caregiver is one of the most valuable tools the caregiver can provide. Respectful, engaging interactions help increase the understanding of the animal’s needs, both as an individual and within a group, ultimately enabling more relevant and enriched relationships and experiences for all.

## Data Availability

Not applicable.
